# The Development of an Embedded Figures Test for the Detection of Feigned Attention Deficit Hyperactivity Disorder in Adulthood

**DOI:** 10.1371/journal.pone.0164297

**Published:** 2016-10-12

**Authors:** Anselm B. M. Fuermaier, Oliver Tucha, Janneke Koerts, Meryem Grabski, Klaus W. Lange, Matthias Weisbrod, Steffen Aschenbrenner, Lara Tucha

**Affiliations:** 1 Department of Clinical and Developmental Neuropsychology, University of Groningen, 9712 TS Groningen, The Netherlands; 2 Department of Experimental Psychology, University of Regensburg, 93040 Regensburg, Germany; 3 Department of Psychiatry and Psychotherapy, SRH Clinic Karlsbad-Langensteinbach, 76307 Karlsbad, Germany; 4 Section for Experimental Psychopathology and Neurophysiology, Centre for Psychosocial Medicine, University of Heidelberg, 69115 Heidelberg, Germany; 5 Department of Clinical Psychology and Neuropsychology, SRH Clinic Karlsbad-Langensteinbach, 76307 Karlsbad, Germany; University of California Los Angeles, UNITED STATES

## Abstract

**Objectives:**

It has been shown that an increasing number of adults deliberately feign attention deficit hyperactivity disorder (ADHD), which demonstrates the need for new tests designed to detect feigned ADHD.

**Methods:**

An Embedded Figures Test (EFT) was developed for the detection of feigned ADHD in adulthood. EFT performance of 51 adults with ADHD was compared to the performance of 52 matched healthy individuals, as well as to 268 undergraduate students who were randomly allocated in a simulation design to one of four experimental conditions, i.e. a control group, a naïve simulation group, a symptom-coached simulation group or a test-coached simulation group. Furthermore, an independent sample of 11 adults with ADHD as well as a sample of 17 clinicians experienced in the work with adults with ADHD were assessed for further validation of the EFT.

**Results:**

The EFT was relatively easy to perform for both patients with ADHD and healthy comparisons as shown by low error rates and non-significant group differences. However, simulation groups differed from patients with ADHD by significant and large effects. An EFT index for the prediction of feigned ADHD was derived based on logistic regression coefficients. Receiver Operating Characteristics (ROC) demonstrated good classification accuracy of feigned ADHD relative to ADHD (AUC = 94.8%), i.e. high sensitivity (88%) and specificity (90%).

**Conclusions:**

This study supports the utility of the EFT for the detection of feigned adult ADHD.

## Introduction

The diagnostic assessment of attention deficit hyperactivity disorder in adulthood (ADHD) comprises a complex clinical evaluation, including a variety of assessment tools (e.g. rating scales, interviews, neuropsychological tests) and multiple sources of information (self- as well as informant reports) [1–4]. The complexity of the diagnostic process is emphasized by conclusive findings indicating that a considerable number of individuals self-referred for clinical evaluation of ADHD exaggerate or feign symptoms (i.e. malingering) [5–8]. High base rates for feigned or grossly exaggerated ADHD symptoms have been estimated in particular for college students presenting for clinical evaluation, with rates ranging from 25 to 48% [8]. These individuals might be motivated to be diagnosed with ADHD by external incentives, such as access to stimulant medication as a cognitive enhancer or for recreational reasons, access to social welfare benefits (e.g. because of unemployment), diminished criminal responsibility (e.g. trying to excuse own misconduct (e.g. stealing, or drug use) by impulsive symptoms associated to ADHD) or advantages in the academic context (e.g. awarded extra time for assignments and exams) [6,9–11]. There is a strong societal interest in preventing false-positive (i.e. undetected feigning) diagnoses of ADHD if one considers the manifold negative consequences of unjustified diagnoses. These consequences include the substantial costs of unnecessary assessments and treatments, unjustified usage of limited medical resources, passive support of drug-trafficking and drug-abuse, damaging of public confidence in effective therapies, and disadvantage for those individuals who do not feign ADHD (e.g. in a competitive academic environment when individuals not feigning ADHD do not get awarded extra time for exams).

In the light of the many negative consequences of false-positive diagnoses of adult ADHD, it is not surprising that a number of studies explored the usefulness of standard measures used in clinical practice to distinguish between feigned and genuine ADHD (for comprehensive reviews on this topic see [12,13]). Standard measures used in clinical practice (e.g. symptom rating scales and neuropsychological tests), although not aimed at the detection of feigning, have the advantage that they are routinely used for diagnostic purposes and therefore do not necessarily require additional time and resources at clinical assessment. However, studies exploring the utility of clinical standard measures in detecting feigned adult ADHD revealed that most individuals feigning ADHD scored in a believable range on these tools and produced profiles that resembled those of genuine patients with ADHD, e.g. [5,7,8,11,14–21]. Moreover, even in the case of a significant group difference between healthy individuals instructed to feign ADHD and genuine patients with ADHD, individual scores of those instructed to feign were still in a believable range so that feigning could not be concluded with certainty from questionnaire responses and test scores.

Consequently, research considered the use of tests specifically designed for the detection of feigned cognitive dysfunctions; the stand-alone effort tests (including symptom validity tests, SVT) [7,15,18,22–25]. Although in terms of specificity to genuine ADHD most of these studies reported promising results with good classification rates, the sensitivity to feigned ADHD was poor to moderate for most measures. Insufficient accuracy of current stand-alone effort tests for the detection of feigned ADHD is not surprising if one considers that these tests were originally designed to detect feigned cognitive dysfunction following acquired brain damage, but not to for the detection of feigned ADHD.

These findings underline the urgent need for the development of new stand-alone effort tests specifically designed for the detection of feigned ADHD. A suitable test for this purpose may follow the principles of an Embedded Figures Test (EFT) [26]. In an EFT, participants must determine whether a simple geometric shape composed of a few lines (e.g. a triangle) is embedded inside a more complex figure composed of many intersecting lines. In order to succeed in such a test, participants must discriminate items from their surrounding context, i.e. disregard the whole image and focus on the detail of the shapes. Even though such a test appears to be an attention test to participants, it is rather a visuospatial perceptual task measuring the ability to suppress Gestalt drive (i.e. central coherence) [27–29]. Studies on individuals with neurodevelopmental disorders have provided evidence for a weak central coherence of patients with autism, which makes them superior to typically developing individuals in variants of EFTs that require the detection of local stimuli embedded in complex figures [30,31]. Individuals with ADHD are, despite their deficits in inhibitory control, presumably not affected by weak central coherence and have been shown to perform comparably to healthy individuals on an EFT task [32]. Hence, an EFT might be a promising means to detect feigned attention deficits in the clinical evaluation of ADHD, as it appears to be a cognitively demanding task requiring high levels of selective attention, but is in fact easy to accomplish for most individuals, including patients with neuropsychiatric conditions such as ADHD. Individuals feigning ADHD may overestimate the difficulty of the task and may show an overly poor performance in the attempt to match the performance that they expect from individuals with ADHD. By this, individuals feigning ADHD may be distinguishable from compliant test takers, including healthy individuals as well as patients with genuine ADHD. Similar principles are employed by some of the already established and validated effort tests, which are used for the detection of feigned cognitive dysfunctioning following brain injury, such as the Dot Counting Test (DCT) [33] or the b-Test [34]. These tests rely on skills that are largely preserved in all but the patients with the most severe brain injury, and do thus result in overly poor performance in individuals with low test-taking effort.

Furthermore, the development of an EFT that is sensitive in detecting feigned ADHD might benefit from also considering practice and transfer effects. Studies using EFTs in healthy individuals demonstrated that the repetition of a task with the same stimuli results in practice effects, whereas transfer effects are the consequence of the repetition of a task (applying same principles) with different stimuli [35]. As the execution of an EFT is not assumed to be cognitively exhausting (in terms of sustained attention requirements), task performance of both healthy individuals and genuine patients with ADHD are expected to increase by prolonged test duration if the same principles and/or stimuli are continuously used. Individuals feigning ADHD, however, may mistakenly assume that the prolonged execution of an EFT is cognitively exhausting and may therefore deteriorate in task performance over time in order to match sustained attention deficits which are known to be characteristic for ADHD [36,37].

The present study describes the utility and sensitivity of an EFT which has been designed for the detection of feigned ADHD by making use of the rationale described above. Healthy individuals were allocated to either a control group requiring them to perform the EFT to the best of their abilities, or to one of three simulation groups asking them to perform the EFT as if they suffered from ADHD. The three simulation groups differed with regard to information they were provided prior to the assessment, as individuals attempting to feign ADHD in a clinical setting may prepare themselves before diagnostic evaluation, e.g. by familiarizing themselves with symptom criteria of ADHD and/or about instruments used at a typical diagnostic assessment. EFT performance of these groups was compared to the EFT performance of a group of patients with ADHD. Classification statistics were calculated in order to explore whether the EFT might be a suitable measure to distinguish between genuine and feigned ADHD. For clinical use, it was aimed to achieve overall accuracy as well specificity of at least 90% [18, 38].

## Method

### Participants

#### Patients with ADHD

Fifty-one adults with ADHD participated in the study (see Table 1 for descriptive information and EFT performance). Patients were referred from local psychiatrists or neurologists to the Department of Psychiatry and Psychotherapy of the SRH Clinic Karlsbad-Langensteinbach, Germany. All patients with ADHD were invited to take part in the study on a voluntary basis. Diagnostic assessments were performed by experienced clinicians associated with the Department of Psychiatry and Psychotherapy and involved a clinical psychiatric interview according to DSM-IV criteria for ADHD as devised by Barkley and Murphey [2] including the retrospective assessment of symptoms in childhood and current symptoms. All diagnoses were made in mutual agreement between at least two clinicians belonging to a diagnostic team experienced in the assessment and treatment of adults with ADHD. The diagnostic assessment also included the identification of objective impairments supporting the diagnosis of ADHD (e.g. evidence derived from school reports, failure in academic and/or occupational achievement) and comprised multiple informants, such as employer evaluation, partner reports or parent reports. Diagnostic veracity of all patients was supported by the identification of objective impairments (n = 51) and multiple informants (n = 31). Moreover, all participants completed two standardized self-report rating scales designed to quantify current and retrospective ADHD symptoms (WURS-K and ASR) [39,40]. All patients scored above the recommended cutoffs on these scales, indicating clinically relevant ADHD symptom severity both at present and retrospectively for childhood. Twenty-two of the 51 patients with ADHD met criteria for ADHD–predominantly inattentive type, one patient met criteria for ADHD–hyperactive-impulsive type, and 28 patients met DSM-IV criteria for ADHD–combined type. Twenty-four patients with ADHD exhibited one or more psychiatric comorbidities, including mood disorders (n = 19), personality disorders (n = 5), anxiety disorders (n = 4), substance abuse disorders (with no substance abuse in the previous six months; n = 2), eating disorders (n = 3), posttraumatic stress disorder (n = 2), and somatoform disorder (n = 1). Patients with ADHD suffering from comorbid psychiatric disorders were not excluded because comorbidity is very prevalent among patients with ADHD and is therefore representative of the clinical picture of this condition [41]. Sixteen patients with ADHD were treated with antidepressant medication at the time of the study because of comorbid disorders. However, none of the patients were currently taking medication for the treatment of ADHD symptoms (i.e. stimulant drug treatment), whereas 9 patients with ADHD reported to have been treated with stimulants in the past. Current treatment with medication for ADHD symptoms (i.e. stimulant medication) was an exclusion criterion as individuals of simulation groups were trying to simulate test performance of patients with ADHD not being treated with stimulant medication. In addition to the EFT, all patients were assessed with a validated and normed neuropsychological test battery as part of their clinical examination. The neuropsychological assessment included routine measures of cognition (see material section for description) that are commonly used in clinical practice for the assessment of cognitive functions of patients with ADHD [42].

**Table 1:** Characteristics of healthy comparison participants and patients with ADHD (first sample).

	Healthy comparison participants (HCG)	Patients with ADHD	
**Descriptives**			
N	52	51	
Age (in years)	34.4±11.4	34.0±11.3	
Gender (female/male)	38/24	21/30	
Intellectual functions (IQ) ^a^	104.9±12.1	102.3±12.4	
**ADHD symptom severity**			
Childhood symptoms ^b^	15.3±9.1	45.7±14.4	
Current symptoms ^c^	11.4±7.1	34.1±9.0	
**Embedded Figures Test (EFT)**			
Block 1, RT (sec)	4.76±2.68	5.61±3.51	
Block 1, Errors	1.67±1.58	1.29±1.40	
	HM = 1.36	HM = 1.08	
Block 2, RT (sec)	4.27±2.25	5.36±2.96	
Block 2, Errors	2.08±2.21	1.90±2.33	
	HM = 1.38	HM = 1.14	
Block 3, RT (sec)	2.39±1.61	3.00±1.86	
Block 3, Errors	1.04±0.99	0.86±1.37	
	HM = 0.95	HM = 0.62	
Block 4, RT (sec)	2.48±1.99	3.04±1.93	
Block 4, Errors	0.94±1.66	1.33±2.14	
	-^d^	-^d^	
**Routine measures of cognition**		**M±SD**	**% percentile** ≤ **10**
Focused attention			
*WAFF–RT (ms)*	-	468.0±121.1	31.1
*WAFF–SD (ms)*	-	159.3±87.0	14.9
*WAFF–Omissions*	-	3.55±4.83	12.8
Vigilance			
*VIGIL–RT (ms)*	-	640.3±114.0	53.3
*VIGIL–Correct reactions*	-	84.1±15.6	64.1
*VIGIL–Incorrect reactions*	-	6.0±5.7	48.9
Response inhibition			
*Go/NoGo commissions*	-	11.7±7.4	12.8
Interference			
*STROOP reading interference*	-	0.11±0.11	21.7
*STROOP naming interference*	-	0.08±0.08	15.2
Cognitive flexibility			
*TMT B (sec)*	-	69.8±22.5	38.3
Figural fluency			
*5PT*	-	28.0±8.40	27.3

^a^ Multiple Choice Vocabulary Test (MWT-B)

^b^ Wender Utah Rating Scale–short version

^c^ ADHD Self-Report Scale; Characteristics are reported in Mean±SD; HM = Huber’s M estimator

^d^ HM could not be calculated because of extremely centralized distribution around the median; Only patients with ADHD performed routine measures of cognition. For each test variable, the percentages of patients scoring within the lowest 10% of test norms are displayed. WAFF = Test of Perception and Attention Functions: Focused Attention (VTS); VIGIL = Vigilance test (VTS); Go/NoGo = Go/NoGo test (VTS); STROOP = Stroop interference test (VTS); TMT B = Trail Making Test part B; 5PT = Five-Point Test (5PT), RT = Reaction time; SD = Standard deviation of reaction time.

After completion of recruitment and assessment of participants as described above, an independent sample of 11 adults with ADHD was recruited (age = 28.0±13.3 years, gender (f/m) = 4/7, IQ = 109.1±17.0, ASR = 25.1±9.1, WURS-K = 32.1±15.0). These patients were requested to perform the Embedded Figures Test (EFT, see materials section) in addition to two established tests of performance validity, i.e. the Test of Memory Malingering (TOMM) [43] and the Dot Counting Test (DCT) [33]. This additional sample of patients with ADHD was recruited in order to examine whether EFT performance as obtained from the first sample of patients with ADHD (who did not complete any established effort tests) can be replicated on an independent sample of patients who additionally completed two established tests of performance validity. The established effort tests were performed to provide objective evidence on the credibility of the second sample of patients. Conclusions on the credibility of the first sample of patients with ADHD (n = 51) can be derived from the comparison of EFT performance between both samples of patients.

#### Healthy individuals

A *healthy comparison group* (HCG) (n = 52) was recruited via public announcements, word-of-mouth and through contacts of the researchers involved. Healthy individuals were selected according to age, gender and intellectual functions, resulting in comparable characteristics to the first sample (n = 51) of patients with ADHD (Table 1). Participants in the ADHD group (first sample) and the HCG did not differ significantly with regard to age (t(101) = 0.16; p = .871), gender (χ^2^(1) = 1.65; p = .198) and intellectual functions (t(93) = 1.03; p = .306). However, as expected, patients with ADHD (first sample) scored significantly higher on self-report scales for both current ADHD symptoms (t(101) = 14.32; p < .001) and retrospective ADHD symptoms (t(100) = 12.78; p < .001).

Furthermore, 268 first-year psychology students (183 female, 85 male) of the University of Groningen, the Netherlands, took part in the study in exchange of course credits. Participants had a mean age of 21.4 years (SD = 2.5 years) and a mean IQ of 100.1 (SD = 9.5). Prior to assessment, these participants were randomly assigned to one of four conditions, i.e. the *control group* (CG) (n = 58; age = 21.5±3.3 years, gender (f/m) = 36/22, IQ = 98.0±9.4), the *naïve simulation group* (NSG) (n = 70; age = 21.3±2.4 years, gender (f/m) = 54/16, IQ = 99.7±9.8), the *symptom-coached simulation group* (SSG) (n = 70; age = 21.4±2.4 years, gender (f/m) = 51/19, IQ = 100.4±8.5), and the *test-coached simulation group* (TSG) (n = 70; age = 21.5±2.0 years, gender (f/m) = 42/28, IQ = 101.7±10.1). Whereas the *control group* was instructed to perform all tests and measures to the best of their abilities, *simulation groups* were asked to perform the tests while pretending to be affected with ADHD (feigning ADHD). The *simulation groups* differed with regard to the additional information they received in preparation of their assessment (see materials and procedure sections).

Descriptive variables did not differ significantly between groups, Wilk’s lambda = 0.946, F(9,545) = 1.40, p = .185, η^2^ = .018. First language of all participants was German. None of the healthy individuals reported a history of neurological or psychiatric diseases and none were taking any medication known to affect the central nervous system. Furthermore, none of the healthy participants endorsed clinically relevant scores of ADHD symptom severity as measured with two standardized self-report rating scales designed to quantify current and retrospective ADHD symptoms (below cutoff scores suggesting clinical significance) [41]. The groups differed with regard to instruction and information they were provided prior to the assessment. This is relevant to increase external validity of this study, as individuals attempting to feign ADHD in a clinical setting may prepare themselves before diagnostic evaluation by gathering information about symptom criteria of ADHD as outlined in the DSM, and/or information about instruments used during a typical diagnostic assessment. The information provided to the various groups of the present study is thoroughly described below in the procedure section.

In addition, another group of 17 healthy individuals was recruited, the so-called *expert simulation group*. All participants of this *expert simulation group* were clinically working psychologists or psychiatrists and were experienced in the diagnosis and treatment of adult ADHD. This expert group was requested to feign ADHD and to perform the EFT as if they suffered from ADHD, in order to bring the EFT into clinical context and to test its utility on a group that is considered to be highly trained and having expert status in the field. This group was included in order to check whether the sensitivity of the EFT is robust against the knowledge of clinicians experienced in the assessment and treatment of adult ADHD. By this, we attempted to prove whether the EFT has clinical utility above the clinicians’ experience. Because if clinicians are not able to feign ADHD convincingly when performing the EFT, or in other words are detected by the EFT as potential malingerers, the EFT may represent a valuable tool for clinicians when feigning is suspected.

### Materials

#### Intellectual functions

Intellectual functions (IQ) were measured using the Multiple Choice Vocabulary Test (MWT-B) [44]. This test consists of 37 lines, each comprising one authentic word and four fictitious words. The participants were required to find the authentic word by underlining it. The MWT-B was suggested to provide a valid measure for premorbid IQ that is relatively insensitive to cerebral dysfunction of individuals [44,45]. In studies on typically developing individuals, the MWT-B was shown to correlate fairly well with global IQ, yielding a median correlation of r = 0.72 in 22 samples [46]. However, it must be noted that the MWT-B has also been criticized for its use as a measure of premorbid IQ in neuropsychological assessments of cognitively impaired patients. For example, it has been shown that the MWT-B systematically underestimates the level of premorbid IQ in case of increasing global cognitive impairment [47]. In the present study, rather homogeneous groups’ characteristics can be expected given that instructed simulators and controls were all recruited via a first year undergraduate program. Nevertheless, we applied the MWT-B to our participants in order to get a rough estimation of intellectual functions, to assure that intellectual functions of all participants are indeed at least in the normal range and to ensure that groups’ characteristics were similar. We opted for a brief IQ assessment over a full assessment because of time constraints. Rather low IQ scores of undergraduate students (Mean IQ around 100) might be explained by the fact that vocabulary skills increase with age in early adulthood and may thus not yet be fully developed in this age. In this context, it must be noted that the MWT-B is not normed according to age groups.

#### Self-reported evaluation of ADHD symptom severity

Childhood ADHD symptoms were self-rated with the short version of the Wender Utah Rating Scale (WURS-K) including 25 items on a five-point scale [40]. Severity of current ADHD symptoms was self-rated with the ADHD self-report scale (ASR) [39] consisting of 18 items on a four-point scale corresponding to the diagnostic criteria of DSM-IV [48]. A sum score was calculated for each rating scale.

#### Embedded Figures Test (EFT)

The EFT comprised 96 items, presented consecutively on a 15” notebook screen using the presentation software E-Prime 2.0. Each item showed simultaneously a complex figure on the left and a simple target figure on the right side of the. In half of the items, the target figure was embedded within the complex figure. The presentation of items was divided into four blocks of 24 items each. Items of each of the four blocks included the same 24 complex figures. Two different target figures were designed, one target figure for Blocks 1 and 3 and another target figure for Blocks 2 and 4. Consequently, Blocks 1 and 2 (i.e. trial 1) were identical to Blocks 3 and 4 (i.e. trial 2), however, presentation order of test items was randomized within all four blocks. The target figure was embedded in twelve of the 24 complex figures per block, always presented at the same spatial location, in the same spatial orientation as well as the same size. This design was employed in order to create a task that may appear difficult to participants, but is actually rather easy to perform for most individuals. Participants were requested to indicate whether the target figure was hidden within the complex figure (by pressing the *Yes* response button on the input device) or not (by pressing the *No* response button on the input device). Participants were explicitly instructed that this was a cognitively complex task requiring high demands on attention and concentration. Before the testing started, a test trial containing three example items was performed. The actual assessment was only initialized if correct answers were given to all example items. The test was self-paced as the subsequent item was presented as soon as a response was given to the current item. Between each block, participants were reminded that the test requires high attentional demands and were requested to maintain high cognitive effort. The mean response time and number of errors per block were registered.

#### Routine measures of cognition (clinical neuropsychological assessment as only applied in the first sample of patients with ADHD)

***Focused attention*** was assessed with the test WAFF (Perception and Attention Functions: Focused Attention) [49] of the Vienna Test System (VTS) [50], a computerized test battery for the measurement of various neuropsychological functions (www.schuhfried.com/vienna-test-system-vts). In the WAFF test, a sequence of visual stimuli against a background of distracting stimuli was presented to participants on a computer screen. Participants were requested to respond to a predefined change in relevant stimuli but to ignore all other types of stimuli. The mean reaction time to stimuli (ms), the standard deviation of reaction times, as well as the number of omissions were registered as measures of focused attention. Internal consistency (Cronbach’s α) of the WAFF ranged between 0.93 and 0.97. Associations to other measures of cognition supported the construct validity (convergent and discriminant validity) of the WAFF.

***Vigilance*** was measured with the computerized test VIGIL of the VTS [50]. In this test, a white dot moved along a circular path (resembling the second hand of an analog clock) in small regular jumps. Occasionally (1 out of 10 jumps), the white dot made a double jump (critical stimulus). The participants were requested to react to this infrequent event of a double jump as quickly as possible with a button press on a response panel. The number of correct responses (to critical stimuli), the number of incorrect responses (to non-critical stimuli, i.e. to events without a double jump) and the mean reaction time (ms) for correct responses were measured. Internal consistency (Cronbach’s α) of the VIGIL ranged between 0.75 and 0.97. The test is described to have high content validity as its design meets all criteria for the operationalization of vigilance.

***Response inhibition*** was measured with a Go/NoGo paradigm (INHIB) of the VTS [50]. In Go/NoGo paradigms, participants are required to distinguish between stimuli requiring a motor response and stimuli requiring the inhibition of a motor response. In this test, a sequence of triangles (80% of the trials) and circles (20% of the trials) were presented on a computer screen. Participants were requested to respond only to the presentation of triangles (Go trials) with a button press on a response panel, but to suppress responses to the presentation of circles (NoGo trials). The task created a prepotency towards responding as the triangle was presented more often (80% of the trials) than the circle. Responding to circles resulted in commission errors which were taken as a measure of response inhibition. Split-half reliability of the Go/NoGo paradigm INHIB is 0.83 (between first and second half of the test). Construct validity at the level of neuropsychological functions was confirmed by factor analytic studies.

***Interference*** was assessed with the Stroop interference test (STROOP) of the VTS [50]. The test consisted of four parts. In the first part, participants were requested to read color words presented in grey color (e.g. the word ‘blue’ displayed in grey color) on a computer screen (reading baseline condition). In the second part, participants were requested to name the color of bars presented on a screen (naming baseline condition). In the third part (reading interference condition), participants were requested to read color words (similar to part one), and in the fourth part (naming interference condition) participants were requested to name the colors in which the words were written (similar principle as part two). However, the color of the words of part three and four were always different from the color names (e.g. the word ‘blue’ displayed in red color). A reading interference score (difference of the median reaction time of the reading interference condition and the reading baseline condition) and naming interference score (difference of the median reaction time of the naming interference condition and the naming baseline condition) were calculated. Internal consistency (Cronbach’s α) of the STROOP was found to be 0.96 or 0.97, respectively. Convergent and discriminant validity was supported by studies showing associations to other measures of cognition.

***Cognitive flexibility*** was assessed with the Trail Making Test (TMT) [51]. The Trail Making Test consisted of two parts. Part A required participants to draw a line, as fast as possible, between numbers in ascending order. Part B consisted of numbers and letters, and required participants to switch attention between both concepts by drawing a line alternately between numbers and letters in ascending order. For each subtest, the time needed for completion was registered. The performance on part B was used as a measure of cognitive flexibility. Reliability coefficients of the TMT varied considerably, with most above .80. Studies of convergent and discriminant validity at the level of neuropsychological functions provided evidence that the TMT part B is sensitive to cognitive inflexibility [52].

***Figural fluency*** (nonverbal) was measured with the Five-Point Test (5PT) [53,54]. The test consisted of 40 five-dot matrices presented on two papers. Matrices were given in only one configuration of symmetrically and identically arranged dots (identical to the five-dot arrangement on a dice). Participants were required to produce as many different figures as possible within a time period of 2 minutes by connecting the dots within each rectangle within a 2-minute time limit. Participants were instructed that not all dots had to be used. However, participants were requested to complete the task by following two rules, i.e. no figures were to be repeated, and only straight lines between dots were permitted. The number of unique designs created was used as a measure of figural fluency. Interrater reliability (ICC) of the 5PT was excellent, ranging from .998 to 1.0. Test-retest reliability was found to be .77 for a retest period of one minute. Associations to other measures of cognition supported the construct validity of the 5PT.

#### Tests of performance validity

Two established ***effort tests*** were used in the present study, i.e. the Test of Memory Malingering (TOMM) [43] and the Dot Counting Test (DCT) [33]. The TOMM is a visual recognition test consisting of two learning trials and a retention trial. The DCT is a short test requesting participants to visually perceive patterns of dots and use elementary counting and multiplication skills. Both tests measure skills that are assumed to be preserved in most individuals with cognitive dysfunction. Test performance of individuals is interpreted based on the test performance that is expected from individuals with comparable etiology who show normal effort, as indicated by results of a range of ‘normal effort’ diagnostic groups. Thus, the TOMM and the DCT aim to distinguish between credible and suspect effort.

### Design and procedure

#### Assessment of patients with ADHD

All patients with ADHD (first and second sample) were tested individually and received no reward for participation. Written informed consent was sought from all participants prior to the assessment. It was pointed out to patients that all data collected in the research project will be analyzed anonymously and will not affect clinical assessment and treatment.

The first sample (n = 51) of patients with ADHD was assessed with a comprehensive battery consisting of self-report questionnaires, interviews, the EFT, and routine measures of cognition (clinical neuropsychological assessment). For the purpose of the present study, only a selection of measures will be considered. The total duration of the assessment of the first sample of patients with ADHD was about 2.5 hours. The assessment was split into two parts, divided by a break to recover from possible fatigue. The study was conducted in compliance with ethical standards of the Helsinki Declaration and was approved by the local institutional ethical committee (Medical Faculty of the University of Heidelberg, Germany).

After completion of recruitment and assessment of patients with ADHD as described above, the independent sample of patients with ADHD (second sample, n = 11) was assessed by requesting them to perform the EFT in addition to two established effort tests, i.e. the TOMM [43] and the DCT [33].

#### Assessment of healthy participants

All healthy participants were tested individually in a quiet laboratory with the exception of the *expert simulation group* who were assessed individually in a quiet office room at the hospital. At the beginning of the experiment, descriptive and anamnestic information was obtained including age, sex, intellectual functions and self-reported ADHD symptom severity. Furthermore, participants were asked for any history of psychiatric or neurological diseases as well as pharmacological treatment. Descriptive information was obtained from all participants at the beginning of the experiment, before simulation groups were instructed to feign ADHD. The subsequent assessment procedure differed between participants of the various groups (HCG, CG, NSG, SSG, and TSG). The *expert simulation group* was added after all assessments of the other groups (groups of healthy participants and patients with ADHD) have already been completed.

Table 2 presents an overview of instructions given per group, including the type of information participants received. The neuropsychological test battery (routine measures of cognition) was not administered to healthy participants. The assessment of healthy individuals was approved by the Ethical Committee Psychology (ECP) affiliated with the University of Groningen, the Netherlands. All participants gave written informed consent prior to participation.

**Table 2:** Type of information and instruction given per group.

Experimental condition	Scenario to feign ADHD (Vignette)	Information about ADHD symptoms (symptom-coaching)	Information about assessment (test-coaching)	Instruction
Patients with ADHD	No	No	No	Normal behavior
Healthy comparison group (HCG)	No	No	No	Normal behavior
Control group (CG)	No	No	No	Normal behavior
Naïve simulation group (NSG)	Yes	No	No	Feign ADHD
Symptom-coached simulation group (SSG)	Yes	Yes	No	Feign ADHD
Test-coached simulation group (TSG)	Yes	No	Yes	Feign ADHD
Expert simulation group	Yes	No	No	Feign ADHD

Note: Vignette = Description of a scenario in which someone would be motivated to feign ADHD, introducing several benefits related to a diagnosis of ADHD (e.g. financial accommodations, prescription of stimulant medication).

***Participants in the HCG and CG*** were asked to perform all tests applied (including the EFT) to the best of their abilities. Participants received a notification by email on the day before the assessment in which the clinical significance of the study was introduced, but which did not contain information with regard to the aim of the study. The duration of the assessment of these groups was about 50 minutes.

***Participants in the simulation groups (NSG*, *SSG*, *and TSG)*** were asked to perform the EFT while pretending to be affected with ADHD (feigning ADHD). For this purpose, participants in the simulation groups were presented with a vignette, describing a scenario in which someone would be motivated to feign ADHD. Several benefits were introduced in the vignette that may come with a diagnosis of ADHD, e.g. financial accommodations, more flexibility and freedom concerning working hours and deadlines, and the prescription of stimulant medication for improvement of work/academic performance or for recreational use. Information provided in the vignette was restricted to support participants to assume their role but did not contain information about the symptoms or nature of ADHD. Moreover, participants were explicitly instructed to make their feigning of ADHD seem realistic, i.e. by avoiding a very obvious exaggeration. In order to encourage participants to feign ADHD in a believable and realistic manner, participants were informed that the participant who feigned the condition best would be awarded with a top of the range tablet PC. This incentive was implemented because of methodological considerations in malingering research suggesting that an external incentive is an important element when using simulation designs [55,56]. However, due to ethical reasons, the tablet PC was in fact assigned randomly to one of the participants across all conditions, independently of the participants’ test performance. After the vignette had been presented, the additional information (if any) participants received varied depending on which group they were part of (Table 2). The NSG received no further information and no suggestions on how to fake ADHD. The SSG received a description of the diagnostic criteria for ADHD as outlined in the DSM-IV. This approach has been shown in previous studies to provide instructed malingerers with sufficient information to become familiar with the characteristics of ADHD [5,11]. Participants in the TSG were informed about how a diagnostic clinical examination of ADHD is commonly performed, including the use of questionnaires, clinical interviews, and standardized neuropsychological tests. Further, instructions given to the TSG contained information about major characteristics of a neuropsychological test, including its goal (assessment of cognitive functions such as attention or impulse control), design (mostly computerized tests, use of specific input devices), and outcome variables (reaction time to stimuli, number of omission and commission errors). Participants in the TSG were also informed about typical behaviors of patients with ADHD when they are being tested, such as distractibility (which may result in omissions), slower working speed (requiring more time to complete tasks), impulsive behaviors (which may lead to commissions) and high fluctuations in performance over time (larger variability in performance). Participants in the simulation groups received the respective information twice, i.e. first by email on the day before the assessment, and second by the experimenter at the beginning of the assessment. This approach was chosen in order to provide participants with sufficient time to get into the role of feigning ADHD and to think about successful simulation strategies. Participants in the SSG and TSG were requested to respond to a number of questions on the content of information they had been given, in order to ascertain that they indeed read but also understood the information. All participants were able to answer these questions, hence no participant had to be excluded. Finally, participants were requested to start feigning ADHD and to perform the assessment as if they suffered from ADHD. At the end of the assessment, participants were instructed to stop feigning ADHD and were debriefed. All participants in simulation groups indicated that they followed instructions, including instructions to feign ADHD. The assessment of participants in the simulation groups took about 70 minutes.

***Participants of the expert simulation group*** were assessed after completion of the assessments of patients with ADHD and healthy individuals. At the beginning of the assessment, descriptive information was obtained and it was ensured that none of the participants was familiar with the EFT or its principles. Subsequently, participants were presented with the vignette and were requested to feign ADHD and to perform the EFT as if they suffered from ADHD (similar to instructions as presented to the NSG).

### Statistical analysis

Neuropsychological test performance (routine measures of cognition) of the first sample (n = 51) of patients with ADHD was analyzed using descriptive statistics. A neuropsychological impairment was determined on the basis of a commonly accepted categorization of ability levels using test norms (percentile ≤ 10) [52]. EFT performance of all groups was first presented by descriptive statistics. For error variables of the EFT, Huber’s M estimator was calculated because standard deviations were close to or greater than means. EFT scores of the first sample of patients with ADHD and the HCG were then compared using multivariate analysis of variance (MANOVA). Furthermore, multiple univariate analyses were applied to compare EFT performance between the first sample of patients with ADHD, the CG, and all simulation groups (NSG, SSG, and TSG). Pairwise group comparisons were performed by using Tukey’s Honest Significant Difference (HSD) post-hoc tests. A rigorous alpha level was set at .01 to control for the problem of multiple comparisons. Furthermore, interpretations were largely based on effect sizes, as effect sizes indicate the magnitude of an effect independently from the significance and are also considered to be more informative in malingering research compared to statistical significance [57]. Effect sizes were indicated by Cohen’s d and η^2^. The index η^2^ provides information about the proportion of variance which is accounted for by the factor group membership. As described by Cohen [58], η^2^ is a function of the effect size index f. According to Cohen [58], a small effect size (f = .10) corresponds to an η^2^ = .0099, a medium effect size (f = .25) to an η^2^ = .0588 and a large effect size (f = .40) to an η^2^ = .1379. The effect size Cohen’s d was computed for pairwise comparisons between simulation groups and the first sample of patients with ADHD. According to Cohen [58], negligible effects (d < 0.20), small effects (0.20 ≤ d < 0.50), medium effects (0.50 ≤ d < 0.80) and large effects (d ≥ 0.80) were distinguished. As Cohen’s d (and its categorization) was designed to consider relatively small effects as relevant to research [58], it was stressed that more rigorous standards are needed for the assessment of malingering [57]. Rogers [57], therefore, introduced a categorization of effects sizes (Cohen’s d) suitable for malingering research and distinguished between moderate effects (0.75 ≤ d < 1.25), large effects (1.25 ≤ d < 1.50) and very large effects (d ≥ 1.50). In addition, binary logistic regression analysis was carried out in order to determine the validity of the EFT in predicting feigned ADHD relative to true ADHD. All simulation groups (NSG, SSG, and TSG) were collapsed for the purpose of this analysis into one feigning group, as it likely remains unknown in any given assessment context if and how individuals had prepared themselves before the diagnostic evaluation. A group of individuals with different levels of preparation may therefore present with high external validity. A prediction equation (the EFT index) was calculated by summating EFT scores weighted with their logistic regression coefficients in order to determine which combination of EFT scores would be best in predicting feigned ADHD. The accuracy of the newly developed EFT index in detecting individuals feigning ADHD (collapsed group of all simulation conditions, n = 210) relative to patients with ADHD (n = 51) was explored in receiver operating characteristics (ROC). A ROC curve plots the sensitivity against ‘1—specificity’ at each level of the EFT index to predict the criterion (feigned ADHD). ROC analysis allows for determination of an overall accuracy of classification as measured by the area under the curve (AUC), as well as for classification statistics to address specific goals, i.e. high sensitivity or high specificity. Furthermore, classification statistics (sensitivity, specificity, positive predictive value, negative predictive value) were calculated separately for each group of instructed simulators (NSG, SSG, and TSG) in order to explore the effects of coaching, as well as to demonstrate the effect of base rates on predictive accuracy of feigned ADHD.

Test performance of the independent sample of patients with ADHD (n = 11) was analyzed using descriptive statistics. Test scores of the TOMM, DCT, and EFT were presented, together with cutoffs for noncredible performance as suggested in the test manuals and as derived from the present study. Test performance of the *expert simulation group* was also analyzed by means of descriptive statistics. EFT performance was interpreted based on the cutoff as derived from the present study.

## Results

### Neuropsychological functions of patients with ADHD

Table 1 includes a summary of the neuropsychological test performance of patients with ADHD (first sample, n = 51), including group means, within group variability of performance (SD), and the proportion of patients with impairments in each of the functions assessed. The proportion of patients with neuropsychological impairments was determined by calculating the proportion of patients scoring within the lowest 10% of test norms (percentile ≤ 10) [52]. The results indicated attention impairments in about one third (focused attention) to half (vigilance) of patients with ADHD. Moreover, about one third of patients exhibited impairments in cognitive flexibility and/or figural fluency. Only a smaller proportion of patients (13 to 22%) displayed impairments in interference or response inhibition. Cognitive performance level of the present patient sample may be representative for the population of ADHD, as results confirm previous neuropsychological research in showing that the majority of patients with ADHD demonstrate impairments in some, but not all aspects of attention and/or executive control [59,60].

### Group comparisons on the EFT

The comparison (MANOVA) of EFT performance between patients with ADHD (first sample, n = 51) and the HCG indicated a non-significant medium difference between groups, Wilk’s lambda = 0.907, F(8,94) = 1.21, p = .302, η^2^ = .093. However, MANOVA comparing patients with ADHD (first sample) with the CG and simulation groups (NSG, SSG, and TSG) revealed a significant and large effect, Wilk’s lambda = 0.508, F(32,1134) = 7.13, p < .001, η^2^ = .156 (Table 3). Univariate comparisons found significant effects for all variables of the EFT, including *Block 1*, *RT*, F(4,314) = 7.06, p < .001, η^2^ = .083, *Block 1*, *Errors*, F(4,314) = 32.87, p < .001, η^2^ = .295, *Block 2*, *RT*, F(4,314) = 8.28, p < .001, η^2^ = .095, *Block 2*, *Errors*, F(4,314) = 20.15, p < .001, η^2^ = .204, *Block 3*, *RT*, F(4,314) = 5.50, p < .001, η^2^ = .065, *Block 3*, *Errors*, F(4,314) = 32.48, p < .001, η^2^ = .293, *Block 4*, *RT*, F(4,314) = 6.64, p < .001, η^2^ = .078, and *Block 4*, *Errors*, F(4,314) = 22.07, p < .001, η^2^ = .219. Effect sizes ranged from medium to large size. Post-hoc pairwise comparisons (Tukey’s HSD) revealed that patients with ADHD had significantly slower response times than the CG in all four blocks. A comparison between patients with ADHD and simulation groups found that patients needed significantly more time to respond than the NSG (Block 1 and 2), SSG (Block 2), and TSG (Block 2). Furthermore, patients with ADHD made significantly fewer errors than each group of individuals instructed to feign ADHD in each block. Moreover, individuals in the CG made significantly fewer errors than individuals in each simulation group in each block, and responded significantly faster in Block 3 and 4 than the SSG and TSG (Table 3). Effect sizes of group comparisons between patients with ADHD and simulation groups are presented in Table 3. Effect sizes ranged from negligible to large size (Cohen’s categorization), whereas largest effects were found in the number of errors in Block 1 and Block 3. According to the categorization devised by Rogers [56], moderate to large group differences were observed in most comparisons, with a very large effect between patients with ADHD and the SSG in the number of committed errors in Block 3.

**Table 3:** Embedded Figures Test (EFT) performance of patients with ADHD (first sample), control participants, and simulation groups (Mean±SD).

	ADHD	CG	NSG		SSG		TSG	
	M±SD	M±SD	M±SD	ES*	M±SD	ES*	M±SD	ES*
Block 1, RT (sec)	5.61±3.51	3.45±1.50^a^	3.60±2.00^a^	0.73	4.54±2.68	0.35	4.48±2.31	0.40
Block 1, Errors^a^	1.29±1.40	1.31±1.22	4.07±2.80^a^^b^	1.20	4.86±3.01^a^^b^	1.45	5.08±3.15^a^^b^	1.48
	HM = 1.08	HM = 1.13	HM = 3.73		HM = 4.60		HM = 4.70	
Block 2, RT (sec)	5.36±2.96	3.30±1.59^a^	3.17±1.95^a^	0.90	3.81±2.32^a^	0.59	3.98±2.32^a^	0.53
Block 2, Errors^a^	1.90±2.33	1.90±2.17	4.73±3.10^a^^b^	1.01	5.27±3.47^a^^b^	1.11	5.10±3.31^a^^b^	1.09
	HM = 1.14	HM = 1.24	HM = 4.78		HM = 5.20		HM = 5.11	
Block 3, RT (sec)	3.00±1.86	1.80±0.89^a^	2.40±1.93	0.32	2.93±2.07^b^	0.04	3.16±2.14^b^	0.08
Block 3, Errors^a^	0.86±1.37	0.95±1.41	3.59±2.77^a^^b^	1.19	4.81±3.16^a^^b^	1.54	4.51±3.22^a^^b^	1.36
	HM = 0.62	HM = 0.73	HM = 3.38		HM = 4.51		HM = 4.17	
Block 4, RT (sec)	3.04±1.93	1.69±0.85^a^	2.09±1.67	0.53	2.67±1.80^b^	0.20	2.81±1.75^b^	0.13
Block 4, Errors^a^	1.33±2.14	0.91±1.81	3.86±2.97^a^^b^	0.95	4.26±3.12^a^^b^	1.07	4.70±3.73^a^^b^	1.07
	-^c^	-^c^	HM = 3.70		HM = 4.01		HM = 4.46	

CG = Control group, NSG = Naïve simulation group, SSG = Symptom-coached simulation group, TSG = Test-coached simulation group

* Effect sizes (Cohen’s d) of group differences between patients with ADHD and simulation groups

^a^ significant at p < .01 when compared to ADHD

^b^ significant at p < .01 when compared to CG; HM = Huber’s M estimator

^c^ HM could not be calculated because of extremely centralized distribution around the median.

### Prediction of feigned ADHD and development of EFT index

**Fig 1:**
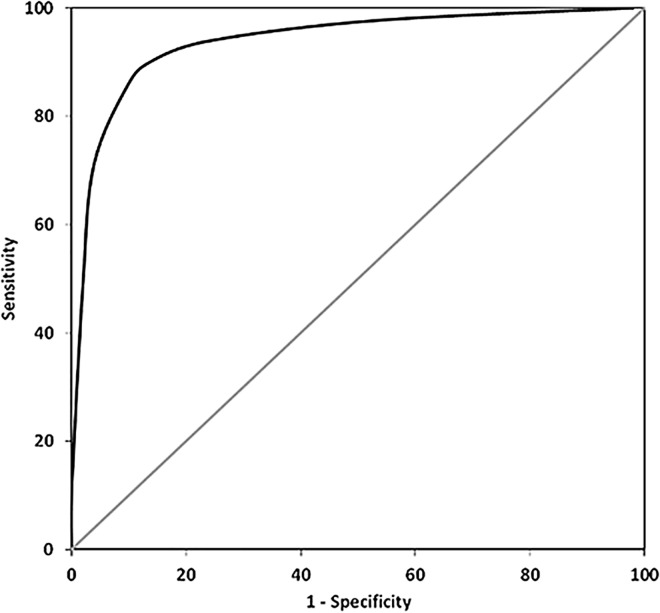
ROC curve indicating diagnostic accuracy of the EFT index in identifying feigned ADHD (all simulation groups collapsed, n = 210) relative to ADHD (first sample, n = 51).

A binary logistic regression model was computed in order to determine the validity of EFT scores (predictors) in predicting feigned ADHD (criterion) relative to true ADHD. A significant model was found for the prediction of feigned ADHD (collapsed group of all simulation conditions, n = 210) relative to ADHD (first sample, n = 51), χ^2^ (8, n = 261) = 140.87, p < .001, explaining 41.7% of the variance (Cox & Snell R^2^). Logistic regression coefficients (Table 4) were used to weight EFT scores of each individual to determine which combination of EFT scores would best predict feigned ADHD. The EFT index was calculated by summing up these weighted EFT scores. A greater value of the EFT index indicates a greater likelihood for feigning ADHD. An inspection of regression coefficients (absolute Beta values) (Table 4) denoted that the number of errors committed in Block 1 and the response time to stimuli in Block 2 and were most informative for differentiating between patients with ADHD and instructed simulators. Differences in sign of coefficients result from the fact that patients with ADHD had larger response times but committed fewer errors compared to participants instructed to feign ADHD. The accuracy of the EFT index for the identification of feigned ADHD relative to ADHD was determined by means of ROC analysis. Data analysis supported the utility of the EFT index in predicting feigned ADHD, AUC = 0.948, SE = 0.015, CI = 0.919;0.977, p < .001. Classification statistics (i.e. sensitivity, specificity, positive predictive value, and negative predictive value) for various cutoffs of the EFT index are presented in Table 5. An inspection of classification statistics aiming to achieve specificity to ADHD of at least 90% [38], results in a suitable cutoff of -0.25 (Table 5). Fig 1 presents a graphical plot of the ROC curve, representing a visualization of the diagnostic accuracy of the EFT index for the identification of individuals feigning ADHD relative to patients with ADHD. Classification statistics for each of the three simulation conditions, i.e. the NSG, SSG, and TSG, are presented in Table 6. The results indicated largely stable classification statistics across the three groups of instructed simulators, although sensitivity was slightly higher for coached groups (SSG and TSG) in comparison to the naïve simulation group.

**Table 4:** Binary logistic regression model to predict feigned ADHD (all simulation groups collapsed; n = 210) relative to ADHD (first sample, n = 51) based on the Embedded Figures Test (EFT) performance.

Predictor variables	B	SE B	Wald	p	Odds ratio
Block 1, RT (sec)	-0.459	0.186	6.104	.013	0.632
Block 1, Errors	0.676	0.207	10.699	.001	1.966
Block 2, RT (sec)	-0.776	0.253	9.411	.002	0.460
Block 2, Errors	0.160	0.140	1.299	.254	1.174
Block 3, RT (sec)	0.342	0.292	1.370	.242	1.408
Block 3, Errors	0.612	0.226	7.347	.007	1.844
Block 4, RT (sec)	0.604	0.368	2.694	.101	1.829
Block 4, Errors	-0.074	0.156	0.228	.633	0.928

**Table 5:** Classification statistics for the identification of instructed simulators (all simulation conditions collapsed, n = 210) relative to patients with ADHD (first sample, n = 51) for various cutoffs of the EFT index.

Cutoff	Sensitivity	Specificity	PPV	NPV
**-1.0**	93.8	72.5	93.4	74.0
**-0.75**	93.3	82.4	95.6	75.0
**-0.50**	90.0	86.3	96.4	67.7
**-0.25**	**88.1**	**90.2**	**97.4**	**65.7**
**0.00**	85.2	90.2	97.3	59.7
**0.25**	83.3	96.1	98.9	58.3
**0.50**	81.4	96.1	98.8	55.7
**0.75**	79.5	96.1	98.8	53.3
**1.0**	78.6	96.1	98.8	52.1

PPV = Positive Predictive Value, NPV = Negative Predictive Value

Classification statistics at the proposed cutoff are indicated in bold.

**Table 6:** Classification statistics for the identification of instructed simulators, separately for each of the three simulation groups, relative to patients with ADHD (first sample, n = 51).

	ADHD–NSG				ADHD—SSG				ADHD—TSG			
Cutoff	Sens.	Spec.	PPV	NPV	Sens.	Spec.	PPV	NPV	Sens.	Spec.	PPV	NPV
**-1.0**	92.9	72.5	82.3	88.1	91.4	72.5	82.1	86.0	97.1	72.5	82.9	94.9
**-0.75**	92.9	82.4	82.3	89.4	90.0	82.4	87.5	85.7	97.1	82.4	88.3	95.5
**-0.50**	90.0	86.3	90.0	86.3	88.6	86.3	89.9	84.6	91.4	86.3	90.1	88.0
**-0.25**	**85.7**	**90.2**	**92.3**	**82.1**	**87.1**	**90.2**	**92.4**	**83.6**	**91.4**	**90.2**	**92.8**	**88.5**
**0.00**	84.3	90.2	92.2	80.7	87.1	90.2	92.4	83.6	84.2	90.2	92.2	80.7
**0.25**	80.0	96.1	96.6	77.8	85.7	96.1	96.8	83.1	84.2	96.1	96.7	81.7
**0.50**	77.1	96.1	96.4	75.4	84.3	96.1	96.7	81.7	82.9	96.1	96.7	80.3

NSG = Naïve simulation group, SSG = Symptom-coached simulation group, TSG = Test-coached simulation group; Sens. = Sensitivity, Spec. = Specificity, PPV = Positive Predictive Value, NPV = Negative Predictive Value; Classification statistics at the proposed cutoff are indicated in bold.

Furthermore, an additional sample of 11 patients with ADHD was recruited and requested to perform the EFT in addition to two established effort tests, i.e. the TOMM and the DCT (Table 7). This additional sample of patients with ADHD was recruited in order to examine whether results as obtained from the first sample of patients with ADHD (who did not complete any established effort tests) can be replicated on an independent sample of patients who also completed two established tests of performance validity. Thus, the credibility of the first sample of patients with ADHD can be explored by the comparison of EFT performance between both samples of patients. Test results of the TOMM and DCT indicated that ten patients with ADHD of the second sample may have shown credible performance, whereas suspect effort was suggested for one patient with ADHD (patient #7). The EFT agreed with this classification of test performances into credible and suspect effort in all eleven cases, i.e. indicating credible performance in ten cases and suspect effort in patient #7 (Table 7). Inspecting EFT performance of both patient groups, it can be observed that the independent sample of patients with ADHD yielded similar scores (-1.74±2.68) than the original sample of 51 patients with ADHD (-2.26±2.14), which is underlined by a nonsignificant nonparametric group comparison (U = -0.396, p = .692). More importantly with regard to the present context, the EFT index as derived from the present study correctly identifies ‘credible’ and ‘noncredible’ test performance (as determined by effort tests TOMM and DCT) of all 11 patients with ADHD, i.e. credible effort in ten cases and noncredible effort in one case.

**Table 7:** Test performance of the independent group of 11 adults diagnosed with ADHD on established effort tests (TOMM and DCT) and the EFT.

Patient with ADHD	TOMM Trial 1	TOMM Trial 2	TOMM Retention	DCT E-score	EFT index
**#1**	50	50	50	8	-2.06
**#2**	47	50	50	11	-0.95
**#3**	50	50	50	13	-0.89
**#4**	48	50	50	13	-5.23
**#5**	49	50	50	13	-4.21
**#6**	50	50	50	13	-0.37
#7*	37	45	40*	14*	4.51*
**#8**	44	50	45	10	-0.80
**#9**	50	50	50	11	-1.29
**#10**	48	50	50	10	-3.60
**#11**	50	50	50	8	-4.29

* indicates low effort according to the cutoffs as provided by test manuals (TOMM and DCT) or as suggested in this study (EFT): TOMM = Test of Memory Malingering, suspect effort if TOMM Trial 2 or TOMM Retention score < 45; DCT = Dot Counting Test, suspect effort if DCT E-Score > 13 (based on normal effort group ‘Depression’); EFT = Embedded Figures Test, suspect effort if EFT index > -0.25.

Finally, EFT performance of the *expert simulation group* as presented in Table 8 shows that none of the experts were able to realistically feign ADHD and to produce EFT scores that one would expect from genuine patients with ADHD. EFT scores of all 17 experts were clearly above the cutoff as derived from the present study (-0.25) indicating noncredible cognitive performance.

**Table 8:** Characteristics and EFT performance of the expertsimulation group.

Expert #	Age (years)	Gender (f/m)	EFT index
**#1**	32	m	6.42
**#2**	30	f	5.35
**#3**	37	f	15.70
**#4**	34	f	4.23
**#5**	49	m	4.78
**#6**	26	f	4.93
**#7**	46	m	3.54
**#8**	26	f	9.14
**#9**	63	f	0.21
**#10**	26	f	6.52
**#11**	28	f	1.80
**#12**	36	f	4.39
**#13**	29	f	1.07
**#14**	28	f	0.71
**#15**	28	f	6.68
**#16**	31	m	3.63
**#17**	34	f	5.73

EFT = Embedded Figures Test, suspect effort if EFT index > -0.25.

## Discussion

The EFT developed for this study was relatively easy to perform for individuals with ADHD and healthy comparisons, as demonstrated by low error rates in both groups (about 6 errors out of 94 items) and a non-significant difference between groups. This is important to stress considering that a proportion of patients with ADHD were found to be impaired in attention and executive control, as indicated by poor performances in measures of cognition that are routinely applied in clinical neuropsychological evaluation. These cognitive impairments did obviously not affect the performance on the EFT. Furthermore, even though EFT performance of patients with ADHD did not differ significantly from healthy comparisons, an exploration of descriptive statistics revealed that patients tended to have longer response times but made fewer errors compared to the HCG. This might indicate a more pronounced trade-off speed for accuracy of patients with ADHD compared to healthy individuals. An inspection of EFT scores across blocks further revealed that both patients with ADHD and healthy comparisons clearly improved performance over time (Block 3 and 4 compared to Block 1 and 2), indicating the presence of practice effects by repetition of the task with the same stimuli [35]. Transfer effects (comparing performance of Block 1 with 2, as well as Block 3 with 4), however, could not be observed in the present data, which might be explained by a more difficult identification of the second target figure (used for Block 2 and 4) compared to the first target figure (Block 1 and 3), when hidden in a complex environment (i.e. the complex figure). Based on the present results, it can be concluded that the design of the EFT largely fulfills the requirements as outlined in the introduction and its principle may be well suited for the detection of feigned attention deficits in the clinical evaluation of ADHD.

Group comparisons of EFT performance between experimental conditions revealed that individuals instructed to feign ADHD performed significantly worse than control participants, as indicated by the accuracy of responses for all simulation groups as well as by the response time for the TSG. These differences demonstrate that healthy individuals assigned to simulation groups were compliant with instructions and altered their normal behavior. Further evidence for successful group manipulations is given as all participants were able to respond to questions regarding simulation instructions, and also indicated after completion of the study that they had followed instructions. A comparison between simulation groups and patients with ADHD showed significant effects in opposite directions on response time and committed errors, as it was found that all groups of instructed simulators committed consistently more errors compared to patients, but had faster response times in some comparisons (i.e. the NSG in Block 1 and 2, the SSG in Block 2). Applying Roger’s [57] categorization of effect sizes; moderate to very large effects with regard to the number of committed errors are indicated, whereas only up to moderate effects with regard to response times were yielded. Interestingly, and contradictory to our expectations, no significant difference was found between any of the three simulation groups on any of the EFT variables. Information about DSM criteria for ADHD (symptom-coaching) or about the procedure of a diagnostic clinical examination (test-coaching) did not benefit instructed malingerers to feign ADHD more realistically. While coaching has been demonstrated to temper flagrant malingering behavior of cognitive dysfunctions following head injury [61–64], the effects of coaching when feigning ADHD have not been studied before, which underlines the novelty and importance of the present findings. Thus, it may be concluded that the preparation of individuals attempting to feign ADHD does not serve them in feigning the condition more credibly during neuropsychological evaluation. Similar results were reported by Dunn and colleagues [55] who explored the effects of symptom information and coaching on the performance on effort tests such as the Word Memory Test [65,66] and the Computerized Assessment of Response Bias-97 (CARB-97) [67]. Instructed malingerers were provided with information about the behavioral effects of brain injury and/or were coached how to successfully “defeat” effort tests. Similar to the present results, Dunn and colleagues [55] found little evidence for the utility of symptom information and coaching in feigning cognitive dysfunctions after brain injury. In fact, the instructed simulators who received information about the behavioral effects of brain injury feigned deficits even more poorly than participants who did not receive this information. This is in line with the results of the present study as depicted in Table 6, showing that coached instructed simulators could even be more accurately identified (sensitivity of 87.1 and 91.4%) compared to naïve instructed simulators (sensitivity of 85.7%). However, it has to be considered that instructed malingerers of the present study were informed about their task per email a day before the assessment. This early introduction to the study provided individuals with sufficient time to prepare and carry out their own research as well as being given standardized instructions as determined by their group assignment. This might have diminished group differences between naïve, symptom-coached and test-coached individuals. Alternatively, it can be speculated that the depth and detail of coaching was not sufficient to adequately prepare coached instructed simulators, resulting in nonsignificant differences between the three groups of instructed simulators. However, the coaching applied accords with the principles of coaching applied in the field of malingering research [57]. Moreover, one may argue that nonsignificant differences between the three simulation conditions emerged from low effort of undergraduate students in both assuming the role of a person with ADHD and performing the assessment as if they suffered from the condition. As a matter of fact, inadequate effort of undergraduate students in studies using neuropsychological tests has been found in a considerable proportion of students [68]. In the present study, we have reasons to believe that the vast majority of students were compliant to instructions and put adequate effort in the assessment, as (1) all students successfully studied information on simulation instructions and coaching (and successfully responded to questions on the content), (2) all participants indicated after completion of the assessment that they had followed instructions and tried to simulate ADHD to the best of their abilities (independently from receiving course credits), and (3) we found a performance pattern across simulation conditions that is similar to the effects of coaching as reported in related research in this field [55]. Finally, students received an incentive for making an effort to feign ADHD, as they were informed that the student who feigns the condition best would be awarded with a top of the range tablet PC. For students who usually have only a limited financial resources, such a tablet PC is of course quite appealing.

More importantly with respect to the aim of the present study, it was examined whether the EFT was useful for the prediction of feigned ADHD. Support for the usefulness of the EFT was given by the logistic regression model demonstrating that the EFT could successfully predict feigned ADHD relative to patients with ADHD. The calculation of the EFT index comprised a summation of weighted EFT scores to find the best combination of variables for the prediction of feigned ADHD. This was done by using a collapsed group of all instructed simulators (NSG, SSG, and TSG), as it would likely not be known in any assessment context if an individual had been coached, and as a mixed group of individuals with different levels of preparation is assumed to represent most realistically the situation at clinical practice. ROC analysis supported the utility of the EFT index for the identification of individuals feigning ADHD, by yielding high overall accuracy (AUC = 94.8%), as well as sensitivity to feigned ADHD of 88.1%, with a specificity to genuine ADHD of 90.2% (cutoff -0.25). The utility of the EFT index for the identification of feigned ADHD can also be concluded from a visual inspection of the ROC plot depicted in Fig 1. High values of sensitivity are particularly promising considering that previous research using established effort tests, including SVTs, reported good specificity, but insufficient or only moderate sensitivity (below 50% for most measures) [7,15,18,22–25]. The EFT as presented in this study could therefore be a valuable contribution to the clinical neuropsychological assessment in order to facilitate the detection of feigned ADHD.

Finally, the clinical utility of the EFT as a valid measure of noncredible cognitive performance of individuals during clinical evaluation of ADHD is supported by results from the *expert simulation group*. All participants of the *expert simulation group* were clinically working psychologists or psychiatrists and experienced in the diagnosis and treatment of adult ADHD. The fact that none of these experts was able to produce a credible score on the EFT that one would expect from someone with genuine ADHD demonstrates the usefulness of the EFT for the clinical setting. On the basis of the present data, it can be concluded that the EFT might be robust to the level of preparation of individuals prior to the assessment, and that the EFT may provide valuable information in the identification of noncredible cognitive performance that might not be obviously visible to the clinician in a standard clinical assessment.

### Limitation and future directions

The EFT index for the identification of feigned ADHD was derived from a sample of patients with ADHD and instructed simulators. Even though sensitivity to detect feigned ADHD was shown on a large sample of individuals that received different levels of coaching, specificity to ADHD was based on one sample of patients only and may therefore require independent replication. A validation of the specificity to genuine ADHD should take different characteristics of patients into account, e.g. comorbidity, medication status, or patients from a specific environment such as college students. A recent study by Williamson and colleagues [24] examined the effect of comorbidity in ADHD on the ability to detect feigning by using established effort tests. The authors concluded that classification rates of feigned ADHD were robust, independently of the presence of comorbid psychiatric disorders of patients with ADHD. A validation study of the EFT for the detection of feigned ADHD may also benefit from the inclusion of a range of conventional and established effort tests used in previous research, in order to directly quantify differences in the diagnostic accuracies of feigned ADHD as achieved by the EFT and conventional effort tests.

As simulation designs are inherently prone to external validity problems, different research designs, such as *known-groups* comparisons, should be used in order to validate the present findings. To assure realistic conditions for simulating ADHD, instructed simulators in the present study received information one day before the assessment, and were motivated by the incentive of a free tablet PC. Nevertheless, a replication of the present findings on individuals seeking clinical evaluation of ADHD, but who are believed to actually feign symptoms, would strengthen implications on the clinical utility of the EFT.

Furthermore, the clinical assessment of patients with ADHD in the present study failed to include a validity measure indicating noncredible effort and symptom reporting. Thus, it cannot be excluded that a proportion of the sample of patients with ADHD may have exaggerated or feigned symptoms, which may have confounded the present analyses. In order to control for this limitation, all diagnoses were made on agreement between at least two experienced clinicians. Moreover, the diagnostic assessment included the identification of objective impairments supporting the diagnosis of ADHD and comprised multiple informants whenever possible. However, in case patients actually did exaggerate or feign symptoms in our sample, this would have resulted in a conservative estimation of the diagnostic accuracy of the EFT for feigned ADHD. Actual group differences between genuine patients with ADHD (showing all adequate effort in the assessment) and instructed simulators would have been even larger than observed, leading to higher classification rates than reported above. Additional evidence on the validity of the present data is given by inspecting test performance of the independent sample of patients with ADHD, in which credible effort was indicated in ten out of eleven cases based on the established effort measures TOMM and DCT. The EFT scores of this sample were comparable to the EFT scores obtained from the original sample of patients with ADHD as shown by a nonsignificant group difference. More importantly, the derived cutoff of the EFT index correctly identified credible performance in ten cases, as well noncredible performance in one case, and thus agrees with the classification of test performances into credible and suspect effort as determined by the established effort tests TOMM and DCT. It can therefore be assumed that even though a performance validity measure has not been included in the assessment of the original sample of patients with ADHD, test results of the original sample have been representative for genuine ADHD and presumably resulted in valid data analysis and conclusions. The inclusion of established effort tests in the assessment of instructed simulators, however, is more complicated as ‘failing’ an effort test cannot be clearly interpreted as low test taking effort in this group, considering that these individuals were instructed to simulate ADHD. Thus we would even expect that participants perform poorly on these tests which implies that low performance is not the consequence of low effort but of a successful simulation strategy. In this case, also, ‘passing’ an established effort test indicates either adequate effort, or otherwise low effort in assuming the role of a person with ADHD and in trying to simulate.

Finally, a concern about many stand-alone effort tests is that they have high face validity as they often present with a distinct character in comparison to classic tests for the assessment of cognitive functions. These effort tests may therefore be easily detected by sophisticated malingerers at clinical evaluation, especially by malingerers who have reviewed adequate information about current effort tests used in clinical practice. The EFT as presented in this study, however, may appear like a classic test for the assessment of selective attention and/or response inhibition, and may therefore be less easily recognized as an effort test by individuals attempting to feign ADHD. Low face validity of the EFT may increase diagnostic sensitivity to feigned ADHD, and may thus support its usefulness for the assessment of adequate test-taking effort. However, face validity of the EFT cannot be explored with the present data, as participants were not asked to identify which tests in the assessment were designed to detect feigning. Furthermore, it is also relevant to explore reliability of the EFT, as it is currently unknown if effort tests such as the EFT are still successful in detecting feigned cognitive dysfunction when applied twice on the same individuals. These issues are to be addressed in future studies when applying the EFT among other tests in a comprehensive and repeated neuropsychological assessment.
